# Are All Ultra-Processed Foods Created Equal? Descriptive Analysis of Nutritional Composition, Nutritional Quality, Energy Density and Hyperpalatability in Relation to Non-Communicable Disease Risk

**DOI:** 10.3390/foods15152651

**Published:** 2026-07-28

**Authors:** Melvin Bernardino, Julia Theresa Regalado, Silvia Gazzin, Claudio Tiribelli, Natalia Rosso

**Affiliations:** 1 Metabolic Liver Disease Unit, Fondazione Italiana Fegato, 34149 Trieste, Italy; ctliver@fegato.it; 2 Department of Life Science, University of Trieste, 34100 Trieste, Italy; julia.regalado@fegato.it; 3 Department of Science and Technology, Philippine Council for Health Research and Development, Taguig 1631, Philippines; 4 Liver-Brain Unit, Fondazione Italiana Fegato, 34149 Trieste, Italy; silvia.gazzin@fegato.it

**Keywords:** Ultra-processed foods, non-communicable diseases, Nutri-Score, energy density, hyperpalatability

## Abstract

Ultra-processed foods (UPFs) are increasingly consumed worldwide, and higher consumption has been linked to higher risk of non-communicable diseases (NCDs). This study examined the nutritional composition and quality, energy density, and hyperpalatability of UPFs available in the Italian food market according to their literature-derived NCD-risk categories. Products were classified into main food groups and subgroups and further categorized according to reported associations with NCD risk: positive, negative, or no reported association. Differences between the main food groups were analyzed in RStudio using non-parametric tests, with results summarized using medians and interquartile ranges. A total of 13,025 UPF products were included in the analysis. UPFs showed substantial variation in nutritional composition, nutritional quality, nutrients-of-concern levels, energy density, and hyperpalatability across the main food groups and literature-derived NCD-risk categories. Snacks represented the largest UPF group and showed the most consistently unfavorable profile, including high median energy, carbohydrate, saturated fat, and sugar contents, a high proportion of Nutri-Score D and E products, and the highest proportion of medium- and high-energy-density products. Overall, 64.3% of UPFs were classified as Nutri-Score D or E, indicating generally lower nutritional quality. However, unfavorable nutritional characteristics were not limited to UPF subgroups reportedly associated with increased NCD risk; some subgroups reportedly associated with lower or no reported NCD risk also contained products with poor nutritional quality, high energy density, or hyperpalatable profiles. These findings suggest that UPFs are not nutritionally uniform and that assessment of UPF-related health relevance should consider food group, subgroup, nutrient profile, energy density, and hyperpalatability in addition to processing classification.

## 1. Introduction

Ultra-processed foods (UPFs) have become increasingly dominant in global food systems and dietary patterns over recent decades [1]. UPF is a categorical term also known as NOVA 4 in the NOVA food framework. This term pertains to food products that are branded, commercially formulated, and made from cheap ingredients extracted or derived from whole foods and combined with additives. These food products are typically manufactured using multiple processing techniques and cosmetic additives designed to enhance palatability, convenience, shelf life, and marketability [2]. Their growing availability and consumption has raised major public health concerns [3], particularly because epidemiological evidence consistently associates high total UPF intake with an increased risk of overnutrition, non-communicable diseases (NCDs) and other health outcomes [2,4]. This accumulating evidence has contributed to a shift in nutritional research from a primary focus on individual nutrients toward broader dietary patterns and, more recently, the degree, purpose, and extent of food processing. This shift has generated considerable debate within the nutrition science community particularly on how effectively the concept of UPFs helps to inform dietary guidelines [5,6,7,8].

Nevertheless, existing data suggest that high UPF consumption is predominantly conceptualized as a uniform dietary category associated with deleterious health outcomes, and robust meta-analytical evidence has demonstrated positive associations between overall UPF intake and the risk of NCDs [3,5,9]. This body of evidence has contributed to the widespread interpretation of UPFs as intrinsically detrimental to health, largely irrespective of the specific type of product consumed. Due to increasing interest, more recent epidemiological and nutritional research has progressively highlighted heterogeneity within UPF subgroups, suggesting that their associations with health outcomes may not be uniform [10]. These findings challenge the assumption of equivalence across all UPF products and indicate that the health effects may vary depending on specific product types, formulation, and nutritional composition. A systematic review of existing observational studies reported that the association between UPF consumption and NCDs varies according to the specific UPF subgroup considered. These findings highlight the importance of moving beyond a generalized evaluation of UPFs and examining the specific nutritional characteristics that differentiate products associated with higher versus lower health risks.

This evolving body of evidence has consequently prompted further investigation into a key research question: whether all UPF products should be considered equivalent in their associations with health outcomes [11], or whether differential effects exist across distinct subcategories of UPFs. In this context, characterizing UPF products according to their nutritional composition may provide important insight into “why not all UPFs are created equal” and why nutrient profile remains a critical dimension in evaluating the health implications of such food products. Supporting this perspective, a quantitative meta-analysis of nationally representative surveys examining UPF consumption and the nutrient composition of respondents’ diets found that, although the contribution of UPFs to total daily energy intake varies across countries, higher UPF consumption is consistently associated with poorer overall dietary quality. However, it remains important to clarify whether these findings are primarily driven by the degree and nature of food processing itself or by the unfavorable nutritional profiles commonly associated with UPFs. A clearer understanding of this distinction will improve the interpretation of future research in this field. Consequently, treating UPFs as a nutritionally homogeneous category may obscure important differences between products and limit our understanding of the mechanisms underlying their observed associations with disease risk [12].

These considerations are particularly relevant when examining real-world dietary transitions at the population level, especially in countries traditionally characterized by healthier dietary habits, such as Italy. Despite the predominance of unprocessed and minimally processed foods, the contribution of UPFs to total energy intake in Italy has nearly doubled, rising from 12% in 2005 to 23% in 2020 [13]. Furthermore, recent evidence indicates that UPFs currently account for approximately 20% of total daily energy intake among the Italian population [14]. These trends are particularly concerning in the context of the progressive decline in adherence to traditional healthy dietary patterns, especially the Mediterranean Diet (MedDiet) [15], which has long been associated with protective effects against NCDs [16,17,18,19]. Evidence suggest that NOVA food classification cannot always identify suitable food choices under the MedDiet pyramid in the modern packaged food environment [20]. Although the concept of UPFs has gained substantial traction in epidemiological research and public health discourse, its scientific robustness, conceptual boundaries, and reproducibility remain subjects of ongoing debate [21].

As dietary habits increasingly shift toward industrially processed food products, there is a growing need to further investigate UPFs in terms of nutritional composition and their potential impact on health outcomes. The Italian food market includes a wide variety of UPFs spanning multiple product categories, with considerable variability in formulation and nutritional composition [22]. Integrating nutrient profile considerations alongside degree of processing may therefore contribute to a more nuanced interpretation of the relationship between UPF consumption and health outcomes. Although most epidemiological evidence linking UPF subgroups to NCD risk derives from studies conducted in non-Italian populations, particularly in the United States, these studies provide an important insight for investigating the nutritional characteristics of UPFs available in other food environments. Given the differences in food formulations, dietary habits, and regulatory contexts across countries, it remains unclear whether UPFs marketed in Italy share similar nutritional profiles to those associated with adverse or protective health outcomes in epidemiological studies. Therefore, examining the composition of UPFs available in the Italian food market may help clarify whether specific nutritional characteristics could partly explain the heterogeneous associations observed between UPF subgroups and health outcomes. Understanding this matter is particularly relevant in places undergoing rapid dietary transitions, where the consumption of UPFs is increasing alongside a gradual decline of traditional dietary patterns. This is essential for improving dietary recommendations, supporting evidence-based food policies, and guiding consumers toward healthier choices within increasingly processed food environments.

Therefore, the present study examined differences in the nutritional profiles of the main groups and subgroups of UPFs. Specifically, UPF subgroups were classified according to their reported associations with NCD risk: (i) positive associations with NCD risk, (ii) negative associations with NCD risk, or (iii) no reported evidence of association. UPF products were assessed according to (a) nutritional composition, (b) nutritional quality assessed using Nutri-Score, (c) nutrients-of-concern levels evaluated through the multiple traffic light (MTL) system, (d) energy density, and (e) hyperpalatability.

## 2. Materials and Methods

### 2.1. Source of Food Data

Information about energy (kcal) and nutrient composition per 100 g (carbohydrates, protein, total fat, saturated fat, salt, and sugars), nutritional quality measured by Nutri-Score, and the degree of processing based on NOVA food classification of pre-packaged food items was obtained from the Open Food Facts database [23]. It is a collaborative and freely accessible online database that contains nutritional information on food products globally. Given its crowdsourced nature, the use of Open Food Facts was supported by systematic checks of data completeness, before products were included in the final dataset.

This crowdsourced repository is available under the Open Database License (ODBL). This database has been widely used as a data source in previous peer-reviewed studies published in reputable journals, supporting the validity and acceptability of this data acquisition approach [24,25]. The database’s advanced search function was employed to filter products by country of origin, with additional filters applied as needed to focus on relevant food categories, nutrient content and the presence of front-of-pack labeling (FoPL). Products meeting these criteria were retrieved, and detailed information on ingredients, nutrition, and labeling was recorded. Nutritional data were standardized to uniform units (e.g., per 100 g or mL), and food categories and FoPL types were consistently coded to enable comparative analysis. All data cleaning, exclusion, and verification steps were logged to maintain traceability, and the final dataset was reviewed for consistency, plausibility, and completeness prior to analysis.

### 2.2. Identifying and Classifying UPF Products

The database’s advanced search function was employed to filter products according to their level of industrial processing, with a specific focus on identifying UPFs. Classification was primarily based on the NOVA food classification system, which categorizes foods into four groups according to the extent and purpose of industrial processing [26,27]. In this study, products labeled as NOVA group 4 were considered UPFs.

#### 2.2.1. Classification According to Main Food Groups and Subgroups

UPFs were classified according to food groups such as beverages, cereals, composite meals, fats and sauces, meat and fish, fruits and vegetables, dairy products, snacks and plant-based alternatives. This categorization was adapted from the FoodEx2 classification system developed by the European Food Safety Authority (EFSA), which provides a standardized hierarchical framework for describing and grouping foods, beverages, and ingredients [28]. Based on the information from the Open Food Facts database we determined the subgroups with the guidance of the FoodEx2 classification. The detailed list of subgroups under the nine (9) main food groups are shown in Supplementary Material Table S1.

#### 2.2.2. Classification According to Reported Associations with NCD Risk

To examine whether the nutritional characteristics of UPF subgroups differ according to their reported associations with NCD outcomes, UPF subgroups were categorized into three groups according to their reported associations with NCDs, based on evidence from previous epidemiological studies in the literature. We mainly based the categories on a systematic review [29] that included 14 research articles (comprising 12 prospective studies and two secondary analyses of randomized clinical trials) where UPF subgroups were identified as follows: (i) UPF subgroups reportedly associated with increased health risks (positive association with NCDs), (ii) UPF subgroups reportedly associated with lower health risks (negative association with NCDs), and (iii) UPF subgroups with no reported associations with health risks. Given that total UPF consumption has been linked to a higher risk of NCDs, UPF subgroups without reported subgroup-specific health associations were also retained in the analysis, as they remain part of the overall UPF exposure in human diet. UPF products were evaluated according to (a) nutritional composition, (b) overall nutritional quality as assessed using the Nutri-Score classification system, (c) levels of nutrients of concern, including total fat, saturated fat, salt, and sugar, (d) energy density, and (e) hyperpalatability.

### 2.3. Nutritional Information and Quality

#### 2.3.1. Nutritional Composition and Energy Density

Nutritional information of UPF products, including energy (kcal), carbohydrates (g), protein (g), total fat (g), saturated fat (g), sugars (g), and salt (g), was obtained from the Open Food Facts database. All nutrient values were recorded on a per-100 g basis to allow direct comparison between products. Additionally, energy density was expressed as kilocalories per gram (kcal/g). As energy content was reported as kcal per 100 g, energy density was calculated by dividing the reported kcal/100 g value by 100. Foods were classified as having low energy density when they contained ≤1 kcal/g, medium energy density when they contained >1 to <3 kcal/g, and high energy density when they contained ≥3 kcal/g [30].

#### 2.3.2. Nutri-Score

Nutritional quality was determined using the Nutri Score, which is a rating system created by international research teams to assess the nutritional value of food products. The Nutri-Score is based on the Food Standards Agency nutrient profiling model (FSAm-NPS), which calculates an overall nutritional score by balancing unfavorable components, such as energy, sugars, saturated fat, and sodium, against favorable components, such as fiber, protein, and the proportion of fruits, vegetables, legumes, nuts, and selected oils. Based on this score, products are classified into one of five color-coded letter grades: A, B, C, D, or E, ranging from higher to lower nutritional quality [31].

#### 2.3.3. Interpretation of Nutrients of Concern Based on MTL Food Classification

Nutrients of concern such as sugars (g), salt (g), saturated fat (g), and total fat (g) were interpreted using the UK’s MTL food classification system (Table 1 and Table 2) [32].

#### 2.3.4. Hyperpalatable Foods Classification

Hyperpalatable foods (HPFs) were defined based on criteria established in the literature and aligned with a three-cluster nutrient profiling framework. The thresholds were developed using a literature-based, data-driven approach to the nutrient profiles of foods described as highly palatable. Thresholds were defined based on the approximate minimum nutrient values characterizing three observed clusters. These criteria captured 70 of the 75 foods discussed in the literature and were subsequently applied to 7757 solid foods in the USDA Food and Nutrient Database for Dietary Studies [33]. The criteria demonstrated convergent validity by identifying most foods commonly regarded as highly palatable and discriminant validity by largely excluding fresh, minimally processed foods. These criteria were considered suitable for the present study because they provide an objective, standardized, and reproducible classification that can be applied directly using energy, macronutrient, sodium, and product-weight information.

This approach classifies foods according to combinations of macronutrient composition and sodium content, reflecting formulations typically associated with industrial processing. Specifically, HPFs were categorized into three clusters: (a) fat- and sodium-dense products, defined as foods containing >25% of total energy from fat and ≥0.30% sodium by weight; (b) fat- and simple-sugar-rich products, defined as foods providing >20% of total energy from fat and >20% of total energy from sugars; and (c) carbohydrate- and sodium-rich products, defined as foods containing >40% of total energy from carbohydrates and ≥0.20% sodium by weight. Products meeting any one of these cluster criteria were classified as HPFs. This multi-criteria approach allows for the identification of foods with different but nutritionally similar UPF profiles, capturing nutrients associated with poor dietary quality.

### 2.4. Data Analysis

Compiled data summarizing the nutritional components of individual UPF products were imported into RStudio v.2024.12.0+467 [34]. Each food sample was grouped based on risk probability (Group 1, Group 2, Group 3). Initial statistics involved tests for normality using the Anderson–Darling test for snacks and Shapiro–Wilk test for the remaining food groups. A non-parametric test was needed to get *p*-values, so the Kruskal–Wallis test was used. This was done by grouping each UPF first by food group and then by risk probability, and the values used were those of the nutrient components. Following *p*-value computations, the 25th quartile, median, and 75th quartile were obtained to understand the distribution of each nutritional value. Nutritional thresholds were then computed first based on food group, then by risk probability. FSAm-NSP scores were also analyzed based on the risk probabilities and Nutri-Scores within the risk probability groups and food subgroups, and these were used for the percentages. Finally, palatability for all food groups was computed based on the stated parameters. Statistical computations were done with tools such as rstatix v.0.7.2 [35] and nortest v.1.0-4 [36] with data visualized using ggplot2 v.3.5.2 [37].

## 3. Results

### 3.1. UPF Products Included in the Analysis

**Figure 1:**
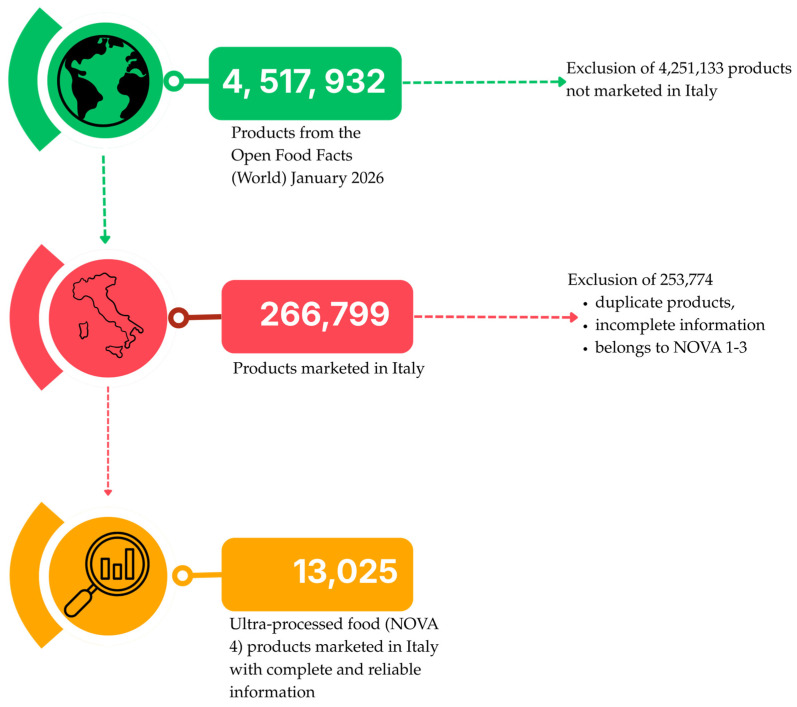
Flowchart of the selection process for UPF products included in the study. Products were obtained from the Open Food Facts database and filtered by market location (Italy), data completeness, and NOVA4 classification, resulting in a final sample of 13,025 products.

As of January 2026, the Open Food Facts (World) database contained 4,517,932 products. Using advanced search filters, we identified 266,799 products marketed in Italy. Products that were not marketed in Italy (4,251,333) were excluded, as well as 253,774 products that were duplicates, contained incomplete information and belonged to NOVA 1, 2 and 3 (unprocessed/minimally processed, processed culinary ingredients, and processed food, respectively). After these exclusions, a total of 13,025 UPF (NOVA 4) products remained for analysis, representing UPFs from Italy with reliable nutritional and labeling information (Figure 1).

### 3.2. Distribution of UPF Products Across Main Food Groups

**Figure 2:**
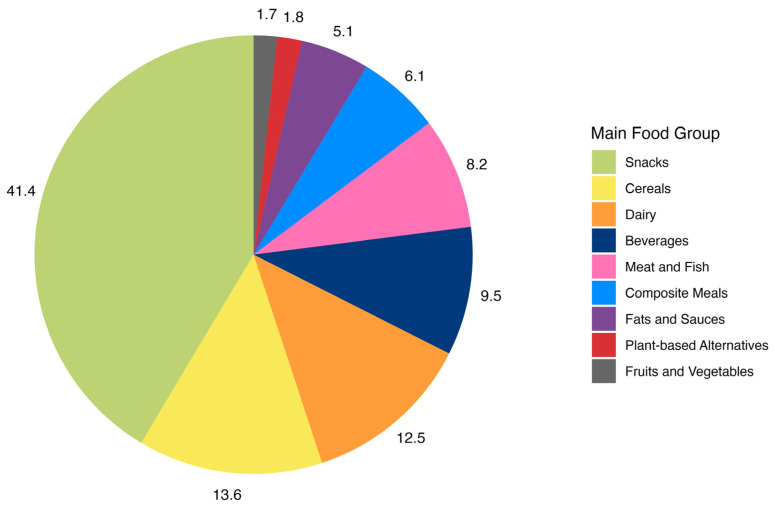
Percentage distribution of UPF products across the main food groups. The pie chart illustrates the relative contribution of each food group to the total UPF sample analyzed.

Snacks comprised the largest proportion of UPF products, accounting for 41.4% (*n* = 5396) of the total identified items. The next most represented groups were cereals, which accounted for 13.6% (*n* = 1776), followed by dairy products at 12.5% (*n* = 1627) and beverages at 9.5% (*n* = 1234). Moderate proportions of UPF products were observed in meat and fish products, representing 8.2% (*n* = 1069) of the total, and composite meals, which accounted for 6.1% (*n* = 800). Fats and sauces contributed 5.1% (*n* = 660). In contrast, the smallest shares were found in plant-based alternatives and fruits and vegetables, accounting for only 1.8% (*n* = 238) and 1.7% (*n* = 225) of UPF products, respectively. The findings showed that snack foods, cereals, and dairy products collectively accounted for more than two-thirds of all identified UPF items (Figure 2).

### 3.3. Main Food Groups and Subgroups According to Their Reported Associations with NCD Risk

The UPF subgroups within each main group were classified into three categories according to the reported direction of their association with NCD risk (Table 3). This grouping scheme was subsequently used to evaluate differences in nutritional composition, Nutri-Score classification, MTL classification, energy density, and hyperpalatability across the three categories. The frequencies and percentages of UPF products within each subgroup are provided in Supplementary Material Table S2.

### 3.4. UPF Assessment Based on Nutritional Composition

#### 3.4.1. Nutritional Composition by Reported NCD Risk

The highest median energy content was observed in Group 3 snacks (nuts) (559 kcal), the highest median protein content in Group 1 processed meat (19.70 g), and the highest median carbohydrate content in Group 2 snacks (60.85 g). These values represent the highest median nutrient concentrations among the food categories. Overall, foods with the highest median energy, protein, and carbohydrate contents were distributed across all three UPF groupings rather than being concentrated within a single group. Differences in energy, protein, and carbohydrate contents were observed across the three UPFs groups (Kruskal–Wallis test, *p* < 0.001 for all variables) (Table 4).

The highest median total fat content was observed in Group 3 snacks (nuts) (40.00 g), the highest median saturated fat content in Group 2 snacks (biscuits, cakes, and chocolate snacks) (8.00 g), the highest median salt content in Group 1 processed meat (2.20 g), and the highest median sugars content in Group 1 snacks (salty and fatty snacks, pastries, and sweets) (29.50 g). These values represent the highest median nutrient concentrations among the food categories. Overall, foods with the highest median total fat, saturated fat, salt, and sugars were distributed across all three UPF groupings rather than being concentrated within a single group. Differences in total fat, saturated fat, salt, and sugar contents were observed across the three UPF risk groups (Kruskal–Wallis test, *p* < 0.001 for all variables) (Table 5).

#### 3.4.2. Nutritional Composition by Main Food Group

The distribution of energy, carbohydrate, and protein contents across different UPF categories using Q1, median, and Q3 values is shown in Table 6. Significant differences were observed among categories for all nutritional variables (Kruskal–Wallis test, *p* < 0.001). Among the main food groups, snacks had the highest median energy content (453 kcal) and carbohydrate content (57.00 g), while meat and fish had the highest median protein content (18.00 g). These values represent the highest median nutrient concentrations among the food groups.

The distribution of nutrients of concern such as total fat, saturated fat, salt, and sugar contents across different UPF categories using Q1, median, and Q3 values are shown in Table 7. Differences in the contents of these nutrients of concern were observed across the UPF categories (Kruskal–Wallis test, *p* < 0.001 for all variables). Among the main food groups, fats and sauces had the highest median total fat content (21.10 g), snacks had the highest median saturated fat content (6.20 g) and sugars content (28.00 g), while meat and fish had the highest median salt content (2.10 g). These values represent the highest median nutrient concentrations among the food group.

Overall, the findings indicate that the nutritional composition of UPFs varies significantly when assessed by both main food groups and reported NCD-risk categories. These results highlight the nutritional heterogeneity of UPFs and suggest that evaluating UPFs according to both food group and nutrient profile may provide a more nuanced understanding of their potential health implications.

### 3.5. UPF Assessment Based on Nutritional Quality

**Figure 3:**
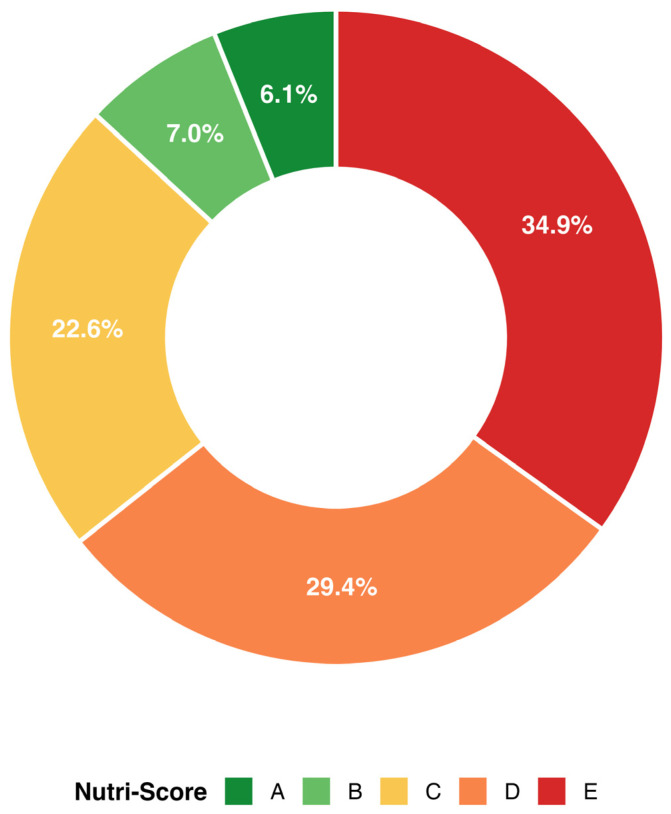
Percentage distribution of UPF products across Nutri-Score categories (A–E). The chart showing the overall percentage distribution of UPFs across Nutri-Score categories. Nutri-Score classifications range from A (dark green, highest nutritional quality) to E (red, lowest nutritional quality).

Among the UPF products analyzed, Nutri-Score E accounted for the largest proportion, representing 4546 products (34.9%), followed by Nutri-Score D with 3829 products (29.4%) and Nutri-Score C with 2944 products (22.6%). In contrast, Nutri-Score B and Nutri-Score A comprised 912 products (7.0%) and 795 products (6.1%), respectively (Figure 3). These findings suggest that the majority of UPF products were characterized by lower nutritional quality according to the Nutri-Score system.

#### 3.5.1. Nutritional Quality by Reported NCD Risk

Across all groups, snacks were the foods most consistently classified as Nutri-Score D and E. This was most evident in Group 2, where the snacks category had 944 products belonging to Nutri-Score D and 1670 products belonging to Nutri-Score E. Group 1 also showed a high burden of Nutri-Score D and E, especially from snacks, meat and fish, fats and sauces, and beverages. The highest Nutri-Score E proportions were observed in snacks and meat and fish, both exceeding 50%. Group 3 had fewer products classified as Nutri-Score E compared with Groups 1 and 2 (Table 8). The findings suggest that Nutri-Score D and E products are not restricted to UPF subgroups with reported increased health risks. However, Group 1 shows the most consistent unfavorable nutritional profile across multiple subgroups, while Group 2 shows an important internal inconsistency driven mainly by snacks. Group 3 appears nutritionally mixed.

**Figure 4:**
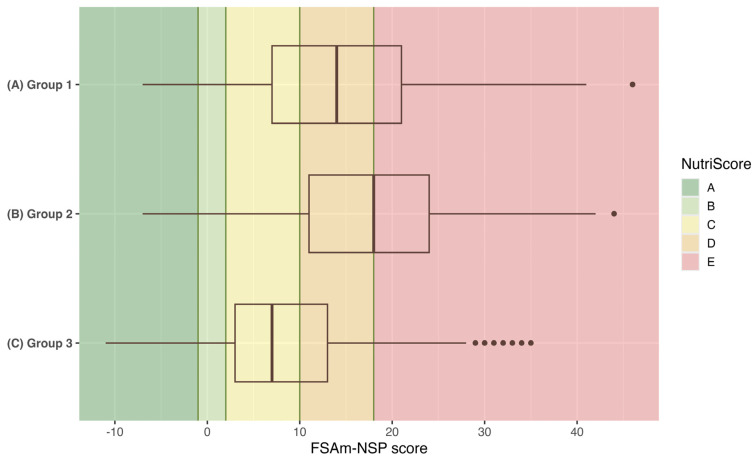
FSAm-NSP scores across groups. Boxplots showing the distribution of FSAm-NSP scores according to UPF NCD-risk groups: (**A**) Group 1 represents UPF food groups associated with increased risk of NCDs, (**B**) Group 2 represents UPF food groups associated with decreased risk of NCDs, and (**C**) Group 3 includes UPF food groups not specifically evaluated for NCD risk in the literature. Boxes represent the interquartile range (IQR), horizontal lines within boxes indicate the median, whiskers show the range of non-outlier observations, and points represent outliers.

**Figure 5:**
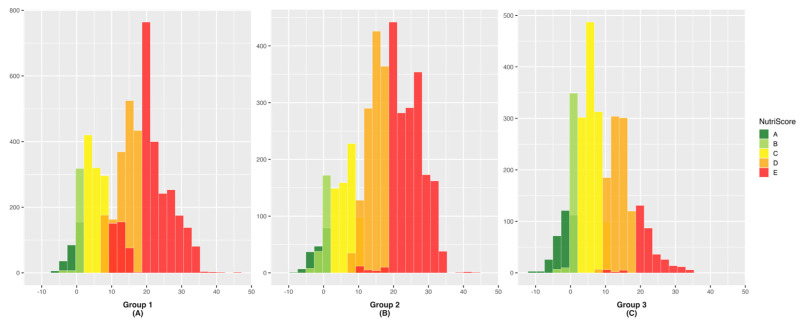
Distribution of Nutri Score classifications across the UPF NCD-risk groups based on FSAm-NPS scores. (**A**) Group 1 represents UPF food groups associated with increased risk of NCDs, (**B**) Group 2 represents UPF food groups associated with decreased risk of NCDs, and (**C**) Group 3 includes UPF food groups not specifically evaluated for NCD risk in the literature. Colors indicate Nutri-Score categories (A–E), where lower FSAm-NPS scores generally correspond to more favorable nutritional quality. The histograms show the variation in nutritional profiles within each UPF risk group.

The distribution of FSAm-NPS scores differed across the three NCD-risk groups, indicating variations in nutritional quality among UPFs. As shown in the boxplot (Figure 4), Group 2 had the highest median FSAm-NPS score, followed by Group 1, while Group 3 had the lowest median score. Since higher FSAm-NPS scores correspond to less favorable nutritional quality, products in Groups 1 and 2 generally exhibited poorer nutritional quality than those in Group 3. The histogram distributions further demonstrate that Groups 1 and 2 were concentrated within the higher FSAm-NPS score range, corresponding predominantly to Nutri-Score D and E categories (Figure 5). In contrast, Group 3 showed a greater proportion of products within the lower and intermediate score ranges, corresponding mainly to Nutri-Score B and C categories, although products spanning all Nutri-Score grades were present. Despite these overall differences, substantial variability was observed within each group. All three groups contained products distributed across multiple Nutri-Score categories, indicating heterogeneity in nutritional quality among UPFs regardless of NCD-risk classification. However, the distribution in Group 3 was shifted towards more favorable FSAm-NPS scores, whereas Groups 1 and 2 showed a greater concentration of products with poorer nutritional quality.

#### 3.5.2. Nutritional Quality by Main Food Group

**Figure 6:**
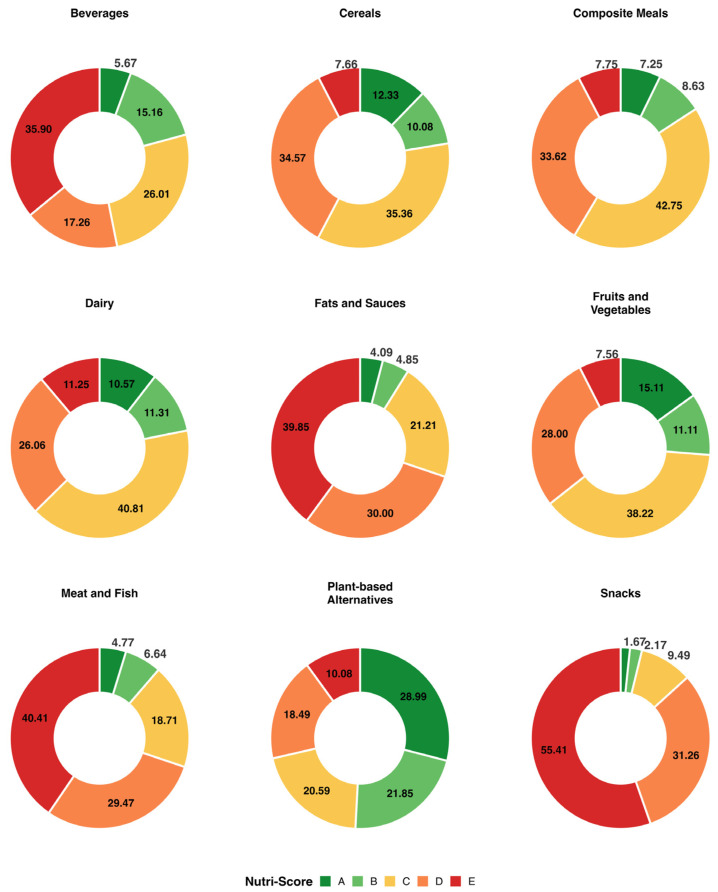
Distribution of Nutri-Score grades across the main food groups. Donut charts illustrate the percentage distribution of foods within each Nutri-Score category (A–E) across food groups. All values presented in the figures are expressed as percentages (%).

This stratified analysis enabled the identification of food categories that contributed most substantially to products with lower nutritional quality such as Nutri-Score D and E as well as those associated with more favorable nutritional profiles such as Nutri-Score A and B. Among all food groups, snacks emerged as the largest source of lower-quality UPFs. More than half of snack products (2985 products; 55.4%) were classified as Nutri-Score E, while an additional 1687 products (31.3%) received a Nutri-Score D. Combined, approximately 86.7% of snack products fell into the two least favorable Nutri-Score categories. Given that snacks also represented the largest food group, they accounted for the greatest absolute number of nutritionally poor products. In contrast, plant-based alternatives exhibited the most favorable nutritional profile among all food groups. Nearly one-third (29.0%; 69 products) achieved a Nutri-Score A, while only 10.1% (24 products) were classified as Nutri Score E. Likewise, fruits and vegetables showed relatively better nutritional quality, with low proportions of products receiving Nutri-Score E (7.6%; 17 products) (Figure 6). Detailed frequencies and percentages for each Nutri-Score category are shown in Supplementary Material Table S3.

**Figure 7:**
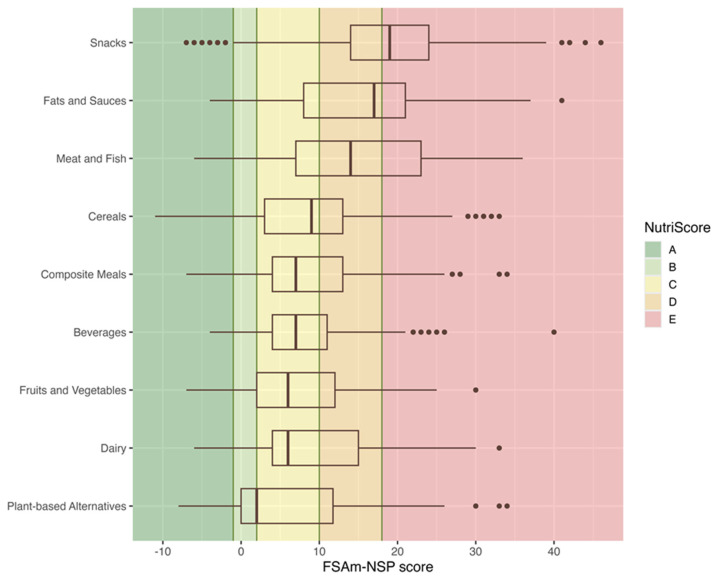
Nutri-Score summary for each food product category. Boxplots showing the distribution of FSAm-NPS scores across different food groups. Higher FSAm-NPS scores indicate lower nutritional quality, while lower scores indicate more favorable nutritional profiles. The shaded background represents the Nutri-Score classification ranges, from A (dark green; highest nutritional quality) to E (red; lowest nutritional quality). The central line within each box indicates the median score, and the box represents the interquartile range (IQR).

Moreover, based on the FSAm-NPS score, most food categories have median scores that fall within the Nutri-Score C and D ranges. Snacks, meat and fish, and fats and sauces tend to have higher median scores and wider score distributions, reflecting lower nutritional quality and greater variability within these categories, while dairy, fruits and vegetables, beverages, and composite meals generally show lower scores (Figure 7).

### 3.6. UPF Assessment Based on Level of Nutrients of Concern

#### 3.6.1. Level of Nutrients of Concern by Reported NCD Risk

**Figure 8:**
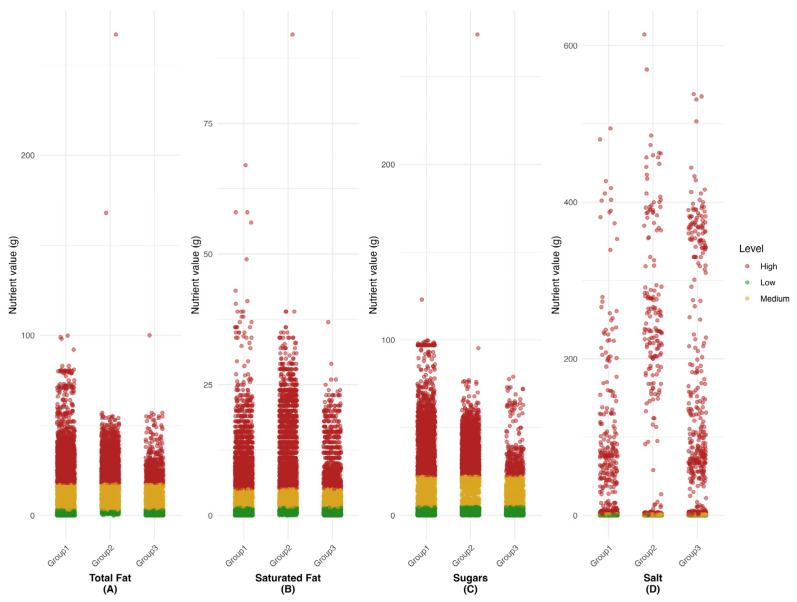
Distribution of food products by MTL nutrient classification across UPF NCD-risk groups. These figures showed the distribution of (**A**) total fat, (**B**) saturated fat, (**C**) sugar, and (**D**) salt values across the main food groups. Each point represents an individual product, while the colors indicate the nutrient classification level (green = low, yellow = medium, red = high).

Across all groups, a substantial proportion of products were classified as high (red) for fat, saturated fat, salt, and sugars, although the extent varied by nutrient (Figure 8). For fat, products in all groups were predominantly classified as high, with Group 1 displaying the widest range of values and several extreme observations. Similar patterns were observed for saturated fat, where Group 2 exhibited the highest values and greatest variability, while Group 3 generally showed lower concentrations. For salt, a large proportion of products across all groups were classified within the high category, with particularly elevated values observed in Groups 2 and 3. Several products displayed extremely high salt content, indicating substantial variability within groups. For sugars, Group 1 showed the greatest spread and highest concentrations, whereas Groups 2 and 3 generally exhibited lower sugar content despite the presence of some high-sugar products. Overall, high sugar classifications were common across all groups.

Furthermore, the products in Group 1 were characterized by a substantial proportion of items classified as high in at least one nutrient of concern. Within this group, the majority of products receiving high nutrients-of-concern ratings were snacks. Fats and sauces contributed the greatest number of products classified as high in total fat, while dairy products accounted for the largest number of high-sugar products. Overall, snacks represented the largest source of high nutrients-of-concern ratings across the food categories assessed. In Group 2, the majority of products receiving high nutrients-of-concern ratings were snacks, particularly for sugars, total fat and saturated fat. In contrast, plant-based alternatives contributed the greatest proportion of products classified as high in salt, followed by cereals. Overall, snacks represented the largest source of high nutrients-of-concern ratings across the food categories assessed, indicating that these products were the primary contributors to elevated levels of sugars, total fat, and saturated fat within Group 2. Group 3 displayed distinct nutrients-of-concern profiles across food categories; within this group, dairy products contributed the largest number of products classified as high in total fat and saturated fat. Meat and fish products accounted for the greatest number of products rated high in salt. Cereals contributed the largest number of products classified as high in sugars. Although snacks showed the highest proportions of products rated high in total fat and saturated fat, this category contained relatively few products. Overall, dairy products, cereals, and meat and fish products were the primary sources of nutrients of concern in Group 3. Detailed frequency distributions of MTL nutrient categories in UPF subgroups belonging to Group 1, 2, and 3 are provided in Supplementary Material Tables S4–S6.

#### 3.6.2. Level of Nutrients of Concern by Main Food Group

**Figure 9:**
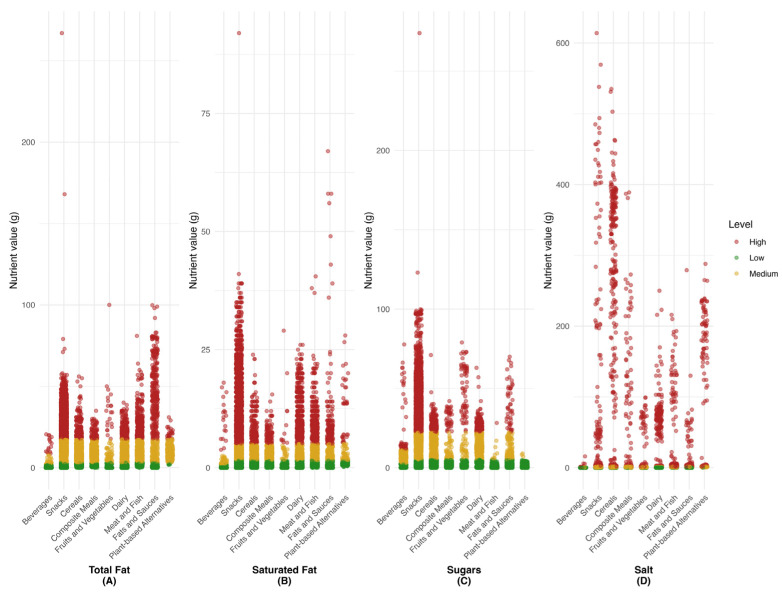
Distribution of food products by MTL nutrient classification across the main food groups. These figures show the distribution of (**A**) total fat, (**B**) salt, (**C**) saturated fat, and (**D**) sugar values across the main food groups. Each point represents an individual product, while the colors indicate the nutrient classification level (green = low, yellow = medium, red = high).

Snacks exhibited the highest concentrations of fat, saturated fat, and sugars, with a large proportion of products classified as high in these nutrients and several extreme outliers. Meat and fish products showed the highest salt levels. Fruits and vegetables and beverages generally displayed lower fat and saturated fat values, although some products had relatively high sugar contents (Figure 9). Overall, the results suggest that snacks, meat and fish, and fats and sauces contribute most substantially to nutrients associated with less favorable nutritional profiles. Detailed frequency and percentage distributions of nutrient levels among the main food groups are shown in Supplementary Material Table S7.

### 3.7. UPF Assessment Based on Energy Density

#### 3.7.1. Energy Density by Reported NCD Risk

**Figure 10:**
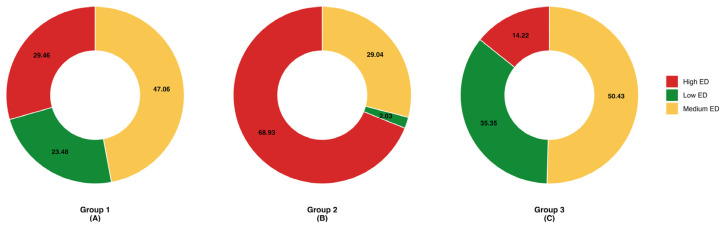
Distribution of UPF products by energy density category across UPF NCD-risk groups. The charts showing the percentage distribution of UPF products according to energy density category within three UPF health-risk groups: (**A**) Group 1 represents UPF food groups associated with increased risk of NCDs, (**B**) Group 2 represents UPF food groups associated with decreased risk of NCDs, and (**C**) Group 3 includes UPF food groups not specifically evaluated for NCD risk in the literature. All values presented in the figures are expressed as percentages (%).

**Figure 11:**
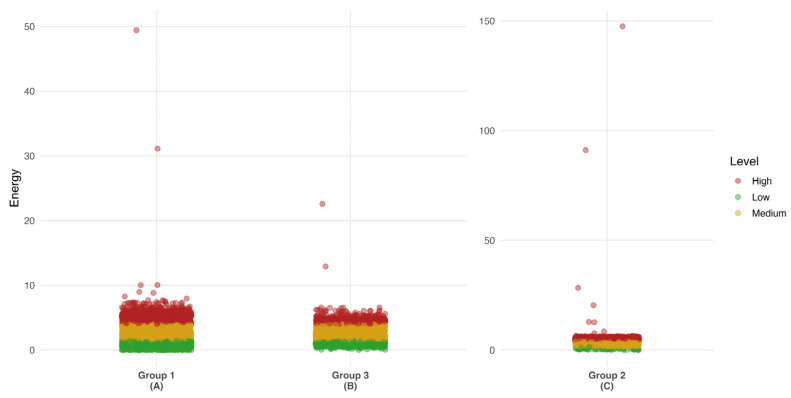
Distribution of energy density values of UPF products across UPF NCD-risk groups. Jitter plot showing the distribution of individual energy density values (kcal/g) of UPF products across three UPF health-risk groups: (**A**) Group 1 represents UPF food groups associated with increased risk of NCDs, (**B**) Group 2 represents UPF food groups associated with decreased risk of NCDs, and (**C**) Group 3 includes UPF food groups not specifically evaluated for NCD risk in the literature. Each point represents a UPF product and is colored according to its energy density category (green = low, yellow = medium, red = high).

Based on the categorical distribution of energy density shown in Figure 10, Group 2 exhibited the highest proportion of high-energy-density products (68.9%), compared with 29.5% in Group 1 and 14.2% in Group 3. In contrast, Group 3 had the highest proportion of low-energy-density products (35.3%) and the lowest proportion of high-energy-density products. Moreover, medium-energy-density products were the predominant category in Groups 1 (47.1%) and 3 (50.4%). Examination of the jitter plot (Figure 11) further reveals differences in the distribution of energy density values among groups. Group 2 displayed the greatest variability and contained the highest energy density values, including several extreme outliers, whereas Group 3 showed a narrower distribution and fewer high-energy-density observations. Group 1 exhibited an intermediate pattern, with a broader distribution than Group 3 and several notable outliers. The frequencies and percentages of UPF products within each subgroup according to level of energy density are provided in Supplementary Material Table S8.

#### 3.7.2. Energy Density by Main Food Group

**Figure 12:**
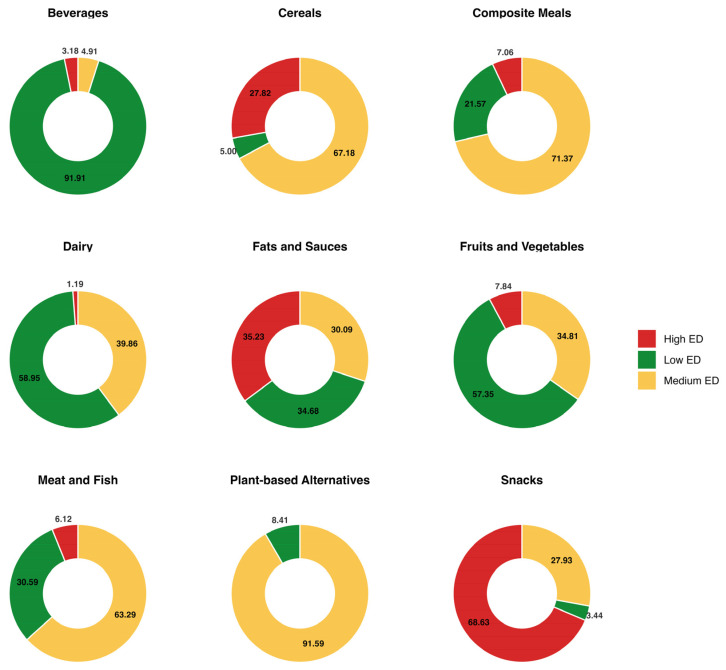
Distribution of energy density categories across food groups. Donut charts show the percentage distribution of low-, medium-, and high-energy-density (ED) foods within each food group. All values presented in the figures are expressed as percentages (%).

The distribution of energy density among food groups shows clear differences in the types of foods (Figure 12). Beverages were predominantly low energy-dense, with 91.9% classified as low ED, indicating that most drinks provide relatively few calories per gram. Dairy products (58.9%) and fruits and vegetables (57.4%) were also largely low energy-dense, reflecting their higher water content and lower calorie concentration. In contrast, snacks showed the highest proportion of high energy-dense foods, with 68.6% classified as high ED and only 3.4% as low ED. This suggests that snack products, which are often UPFs, contribute substantially to calorie intake. Cereals were mainly medium energy-dense (67.2%), although more than one-quarter (27.8%) fell into the high ED category. Similarly, composite meals (71.4%), meat and fish (63.3%), and plant-based alternatives (91.6%) were predominantly medium energy-dense. Overall, the findings indicate that UPF snack foods are the most energy-dense food group, while beverages, dairy products, and fruits and vegetables tend to have lower energy density. Detailed frequencies and percentages for the distribution of energy density categories across food groups are shown in Supplementary Material Table S9.

### 3.8. UPF Assessment Based on Hyperpalatability Criteria

#### 3.8.1. Hyperpalatability Criteria by Reported NCD Risk

**Figure 13:**
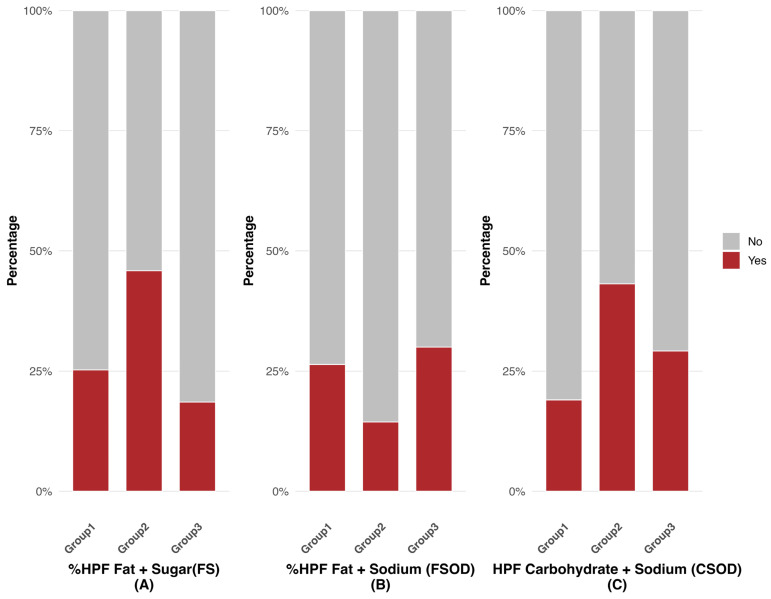
Prevalence of hyperpalatable UPFs according to NCD-risk group. Bars represent the proportion of products meeting (“Yes”) and not meeting (“No”) for the respective HPF criterion within each food group. UPF products classified as HPFs according to the (**A**) Fat + Sugar (FS), (**B**) Fat + Sodium (FSOD), and (**C**) Carbohydrate + Sodium (CSOD) criteria across three UPF subgroup classifications. Red bars represent “Yes” and grey bars represent “No”.

Group 2 exhibited the highest prevalence of HPF products under both the Fat + Sugar (FS) and Carbohydrate + Sodium (CSOD) criteria, with approximately 46% and 43% of products meeting these criteria, respectively. In contrast, Group 3 showed the highest prevalence under the Fat + Sodium (FSOD) criterion, with around 30% of products classified as HPFs. Group 1 displayed intermediate levels of HPFs across all three criteria (Figure 13).

#### 3.8.2. Hyperpalatability Criteria by Main Food Group

**Figure 14:**
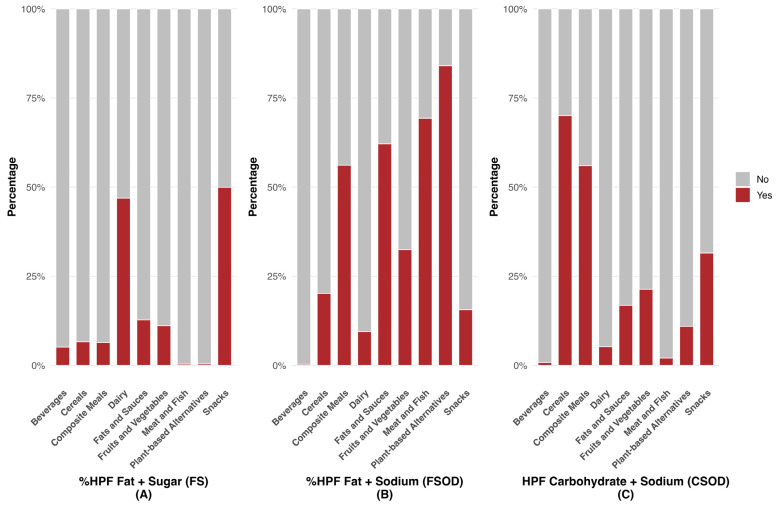
Prevalence of hyperpalatable UPFs across the main food groups. Bars represent the proportion of products meeting (“Yes”) and not meeting (“No”) for the respective HPF criterion within each food group. UPF products classified as HPFs according to the (**A**) Fat + Sugar (FS), (**B**) Fat + Sodium (FSOD), and (**C**) Carbohydrate + Sodium (CSOD) criteria across three UPF subgroup classifications. Red bars represent “Yes” and grey bars represent “No”.

Among the UPFs analyzed, the prevalence of HPFs varied considerably across food groups and according to the nutrient combination criterion applied (Figure 14). Under the Fat + Sugar (FS) criterion, HPFs were relatively uncommon overall and were mainly concentrated in snacks and dairy products, where nearly half of the products met the criterion. In contrast, the Fat + Sodium (FSOD) criterion identified the greatest proportion of HPFs across multiple food groups, particularly plant-based alternatives, meat and fish, fats and sauces, and composite meals, indicating that savory fat–sodium combinations are a dominant feature of many UPFs. Under the Carbohydrate + Sodium (CSOD) criterion, the highest prevalence was observed in cereals and composite meals, with a substantial proportion also found in snacks.

Overall, these findings demonstrate that a considerable proportion of UPFs possess nutrient combinations that meet established definitions of hyperpalatability. While all products were classified as UPFs, the distribution of HPFs was not uniform across food groups, suggesting that certain categories are more likely to contain formulations designed around highly rewarding combinations of fat, sugar, carbohydrates, and sodium. Notably, the FSOD criterion captured the largest share of products, indicating that fat–sodium combinations may be the predominant hyperpalatable profile among the UPFs examined. These results reinforce concerns that many UPFs are formulated not only to be convenient and shelf-stable but also to maximize palatability, which may contribute to increased consumption and potential adverse health outcomes.

## 4. Discussion

UPFs comprise a broad range of industrially formulated products that differ in food category, ingredient composition, and intended use [38]. Under the NOVA framework, UPFs are classified as NOVA group 4, a classification that has contributed substantially to the current understanding of the potential role of industrial food processing in diet quality and health outcomes [27,39,40]. In the present study, we reviewed the literature to identify UPF subgroups reportedly associated with increased, decreased, or no reported association with NCD risk [29]. These subgroups were then matched with UPF products collected from the Open Food Facts database and compared according to nutritional composition, Nutri-Score classification, MTL nutrients-of-concern levels, energy density, and hyperpalatability.

The main finding of this study is that UPFs are not nutritionally uniform. Although all products were classified as NOVA group 4, their nutritional composition, nutritional quality, nutrients of concern profiles, energy density, and hyperpalatability varied substantially across main food groups and literature-derived NCD-risk categories. Previous studies have primarily examined UPFs either through processing-based classifications such as NOVA, through nutrient-profiling systems, or through epidemiological analyses of individual UPF subgroups. However, limited research has directly investigated whether UPF subgroups with different reported associations with NCD outcomes also differ in their product-level nutritional and formulation characteristics. The novelty of the present study lies in integrating these previously separate approaches by comparing literature-derived NCD-association categories using Nutri-Score, MTL nutrients of concern, energy density, and hyperpalatability. This approach advances existing UPF research by examining heterogeneity within NOVA group 4 and by identifying areas of agreement or divergence between epidemiological associations and product-level characteristics, rather than relying exclusively on either processing level or nutrient composition. This finding supports a more nuanced interpretation of UPFs, in which processing-based classification is considered alongside product-level nutritional and formulation characteristics.

### 4.1. Plausibility of the NCD-Risk Groupings

The observed product-level profiles provide partial support for the plausibility of the literature-derived NCD-risk groupings, but they also indicate that these groupings should be interpreted cautiously. UPF subgroups reportedly associated with increased NCD risk generally showed less favorable characteristics across several indicators, including a higher concentration of products in Nutri-Score D and E categories, frequent high levels of nutrients of concern, and the presence of energy-dense and hyperpalatable products. These patterns are biologically plausible because higher levels of sugars, saturated fat, sodium, energy density, and specific hyperpalatable nutrient combinations may contribute to excessive energy intake, poorer diet quality, and adverse cardiometabolic outcomes [41,42]. However, the findings also show that the literature-derived risk categories did not clearly separate UPF products according to nutritional quality or nutrient composition. Some subgroups reportedly associated with lower NCD risk, as well as subgroups with no reported association, still contained products with less favorable nutritional profiles. This was particularly evident for snack-type UPFs, which frequently showed lower Nutri-Score classifications, high levels of sugar, total fat or saturated fat, high energy density, and hyperpalatability profiles. Therefore, subgroup associations reported in observational studies should not be interpreted as evidence that all products within those subgroups are nutritionally favorable.

Contradictory findings in the literature further support this cautious interpretation. Some studies have reported positive associations between UPF desserts or sugary snacks and diabetes, colorectal cancer mortality, or all-cause mortality [43,44,45] whereas other studies have reported inverse or weaker associations for packaged sweet snacks, candy, or chocolate in relation to type 2 diabetes [46,47]. These inconsistent findings may reflect heterogeneity within broad snack categories rather than a beneficial effect of UPF snack consumption. In the present study, snacks emerged as the largest source of lower nutritional quality and showed high concentrations of total fat, saturated fat, and sugars, as well as the highest proportion of high-energy-density products.

In real-world consumption, portion size, frequency of intake, dietary context, and contribution to total energy intake may help explain differences between product-level profiles and epidemiological associations. Snacks are important contributors to energy intake in some dietary patterns [48] and frequent consumption of energy-dense snacks may contribute to higher total energy intake and body weight in adults [49]. Observed inverse or neutral associations in some cohort studies may also be influenced by smaller portion sizes, lower frequency of consumption, replacement foods, residual confounding, or differences in consumer characteristics. Considering the poor nutritional profile of UPF snacks, policies and practices suggest the reduction of portion sizes could help discourage excessive intake of UPFs and reduce their contribution to poor diet quality given the increasing availability of snack-type UPFs in the modern food environment [50]. Therefore, communication of subgroup-specific findings should avoid implying that some UPF categories can exert protective effect or lower the risk. A more appropriate interpretation is that UPF subgroups differ in reported associations and in product-level nutritional characteristics.

Overall, the strength of this approach is that the NCD-risk categories were not imposed independently of the literature but were derived from existing epidemiological evidence and then examined using multiple product-level indicators. However, the present analysis remains descriptive and cannot establish causal relationships with NCD risk. The risk groupings should therefore be interpreted as literature-informed and hypothesis-generating rather than as definitive indicators of disease risk for individual products or subgroups. The findings suggest that UPF subgroups differ nutritionally, but that subgroup-level risk classifications should be interpreted in the context of within-group heterogeneity, portion size, consumption frequency, dietary pattern, and product reformulation.

### 4.2. Nutritional Heterogeneity of UPFs: Evidence from Nutrient Profiling, Energy Density, and Hyperpalatability

Nutri-Scores provided a useful summary measure of nutritional quality in this product-level analysis. In public health settings, Nutri-Scores are intended to help consumers compare the nutritional quality of products and support healthier food choices [51]. In the present study, most UPF products were classified in the less favorable Nutri-Score categories, with 64.3% of products classified as Nutri-Score D or E. This is consistent with previous cross-classification studies showing that UPFs are more frequently represented in lower nutritional quality categories [24,52,53]. This finding is important for positioning the study within the UPF debate. It shows that although the UPF category contains heterogeneous products, the overall product supply remains skewed toward lower nutritional quality. Thus, the conclusion is not that UPFs are generally harmless, but that their nutritional risk is unevenly distributed across subgroups.

Evidence from cohort studies also supports the public health relevance of poorer nutritional quality assessed using nutrient profiling systems. Diets with less favorable Nutri-Score-based profiles have been associated with higher risk of cardiovascular events, myocardial infarction, and stroke [54]. In the present study, MTL nutrients of concern classification complemented the Nutri-Score findings by showing that lower nutritional quality was not driven by a single nutrient. Instead, different UPF groups showed different combinations of high total fat, saturated fat, sugars, or salt. Snacks were particularly characterized by high fat, saturated fat, and sugar levels, whereas meat and fish products showed higher salt levels. This suggests that the nutritional concerns associated with UPFs vary by product category. The assessment of energy density and hyperpalatability provided additional information beyond nutrient composition alone. Previous studies have reported overlap among UPFs, high-energy-density foods, and hyperpalatable foods [55,56]. In the present study, snacks showed the highest proportion of high-energy-density products, while beverages, dairy products, and fruits and vegetables were more commonly low energy-dense. This indicates that UPF status alone does not fully capture the energy-density profile of products.

Hyperpalatability also varied across UPF groups and risk categories. Group 2 showed the highest prevalence of products meeting the Fat + Sugar and Carbohydrate + Sodium hyperpalatability criteria, whereas Group 3 showed the highest prevalence under the Fat + Sodium criterion. These results suggest that hyperpalatable formulations cut across literature-derived NCD-risk categories and may not be confined to UPF subgroups reportedly associated with increased NCD risk. This is relevant because hyperpalatable foods have been linked to higher energy intake, eating beyond fullness, and weight gain in observational and experimental research [57,58,59,60].

Taken together, these findings indicate that UPFs are generally characterized by poorer nutritional quality, but that the UPF category includes products with diverse nutrient compositions and formulation profiles. This is consistent with previous studies showing that UPFs are often higher in energy density, sugars, saturated fat, total fat, sodium or salt, and additives, while being lower in fiber, protein, and some micronutrients compared with less-processed foods [61,62,63,64,65,66,67]. The present study adds to this evidence by showing that even within UPFs, nutritional and product-level characteristics differ substantially across main food groups and subgroups.

### 4.3. Are All UPFs Created Equal?

The question of whether all UPFs are created equal is central to current nutrition science and public health debate. The present findings suggest that they are not. Although all products in this analysis were classified as UPFs, they differed substantially in nutritional composition, Nutri-Score classification, MTL nutrients-of-concern levels, energy density, and hyperpalatability. These differences were observed both across main food groups and across literature-derived NCD-risk categories. The answer provided by this study is therefore qualified: all UPFs are not created equal in nutritional terms, but this does not mean that some UPFs should automatically be considered healthy or protective. The more evidence-based interpretation is that UPFs vary in their nutritional quality and formulation, and these differences may partly explain why epidemiological studies report different associations across UPF subgroups.

This does not mean that UPF classification is unimportant. High overall UPF intake has been consistently associated with adverse health outcomes in epidemiological studies [4,68]. However, the aggregate UPF category may mask important variation across subgroups. Recent evidence suggests that some UPF categories, including processed meats, sweetened beverages, sauces, spreads, condiments, ready-to-eat meals, and some animal-based products, are more consistently associated with higher risk of type 2 diabetes, multimorbidity, cardiovascular disease, or mortality [43,46,47,69,70,71]. By contrast, other UPF categories, such as some breads, breakfast cereals, yogurt products, or plant-based products, may show weaker, neutral, or inverse associations in some observational studies [47,70].

The observed heterogeneity among UPF subgroups may also partly reflect differences in the food matrix, defined as the physical structure and organization of nutrients within a food [72]. The food matrix can influence eating rate, nutrient release, digestion, and metabolic responses; therefore, products with similar nutrient profiles may not necessarily exert the same physiological effects. [73,74]. This may help explain why the nutritional characteristics and reported NCD associations of UPF subgroups were not consistently aligned in the present study. However, food-matrix characteristics were not available in the present database and should be explored in future studies to determine whether differences in food structure and matrix disruption contribute to the observed heterogeneity among UPFs.

The present findings support the interpretation that UPFs differ in product-level characteristics that may be relevant to health. However, these differences should not be interpreted as evidence that some UPFs are inherently protective. Observed subgroup associations may reflect nutrient composition, food matrix, fortification, replacement foods, dietary pattern, socioeconomic factors, or residual confounding. Therefore, the more cautious interpretation is that UPF subgroups are heterogeneous and that their health relevance may depend on product type, nutritional quality, formulation, portion size, frequency of consumption, and role within the overall diet.

### 4.4. Relevance of the Study in the Prevention and Management of NCDs

Although the NCD-risk groupings did not fully distinguish UPF products according to nutrient profile, the findings remain relevant for NCD prevention and management. The study shows that UPFs are heterogeneous and that their potential health relevance cannot be assessed by processing level alone. Conventional indicators such as fat, saturated fat, sugars, salt, energy density, and Nutri-Score provide important information, but they may not fully capture other factors related to UPF consumption, including food matrix disruption, industrial formulation, additives, hyperpalatability, portion size, frequency of intake, and dietary context.

From a public health perspective, these findings support a combined approach. Processing-based classification can identify a broad category of industrially formulated foods that may be relevant to health, while nutrient profiling, energy density, and hyperpalatability criteria can help identify products with less favorable characteristics within that category. Prevention strategies may therefore need to prioritize UPF subgroups that combine poor nutritional quality, high levels of nutrients of concern, high energy density, and hyperpalatable formulations, rather than treating all UPFs as nutritionally equivalent.

This approach is also relevant to metabolic dysfunction-associated steatotic liver disease (MASLD) prevention because MASLD is closely linked with obesity, insulin resistance, type 2 diabetes, and cardiometabolic risk. The present study did not measure liver outcomes; however, it identifies UPF subgroups that combine characteristics relevant to MASLD prevention, including high energy density, high sugar or saturated fat content, lower nutritional quality, and hyperpalatable formulations. These findings support targeted reduction of UPF categories such as snacks, sweetened beverages, processed meats, and fats and sauces within broader dietary strategies that emphasize overall diet quality.

At the same time, the weak separation between some risk categories highlights the need for caution when using subgroup classifications as direct indicators of disease risk. Product-level indicators should not be viewed as substitutes for prospective dietary intake and health outcome data. Rather, they can help generate hypotheses about which UPF subgroups may be more relevant for NCD prevention and which product characteristics should be considered in future epidemiological and intervention studies.

### 4.5. Strengths and Limitations of the Study

This study has several strengths. First, it used product-level data from Open Food Facts to assess pre-packaged UPFs available in Italy across multiple food groups and subgroups. Second, it applied established classification systems, including NOVA, FoodEx2, Nutri-Score, MTL criteria, energy-density thresholds, and hyperpalatability criteria. This allowed a multidimensional assessment of UPFs beyond processing level alone. Third, the study examined UPF subgroups according to literature-informed NCD-risk associations, allowing an exploratory assessment of whether subgroups with different reported health-risk patterns also differed nutritionally. Finally, by combining nutrient composition, nutritional quality, nutrients of concern, energy density, and hyperpalatability, the study provides evidence of nutritional heterogeneity within UPFs and highlights the value of complementing NOVA classification with product-level indicators.

The study also has limitations. It relied on Open Food Facts, a crowdsourced database; therefore, product information may be incomplete, outdated, inconsistently reported, or not fully representative of all UPFs available in Italy. Missing nutrient, ingredient, Nutri-Score, NOVA, or labeling data may have introduced selection bias. The analysis was cross-sectional and product-based, so it did not assess actual dietary intake, portion size, purchase patterns, market share, consumption frequency, or health outcomes. Therefore, it cannot establish causal links with NCD risk. The literature-informed NCD-risk groupings should be interpreted as hypothesis-generating rather than as definitive evidence of risk for individual products or subgroups. Finally, NOVA, Nutri-Score, MTL, energy-density, and hyperpalatability classifications each have inherent limitations and may not fully capture food matrix effects, additives, reformulation, sensory response, or real-world consumption patterns.

## 5. Conclusions

The findings suggest that UPFs are not all created equal. Although all products were classified as UPFs, their nutritional composition, Nutri-Score profiles, MTL nutrients-of-concern classifications, energy density, and hyperpalatability patterns varied substantially across food groups and literature-derived NCD-risk categories. Most UPFs showed less favorable nutritional quality, but this was not evenly distributed across product categories. Snacks, processed meats, fats and sauces, and selected sweetened products appeared to contribute more substantially to unfavorable product-level profiles, whereas some other UPF groups showed comparatively better profiles. Accordingly, the central contribution of this study is to bridge the epidemiological question of whether UPF subgroups differ in NCD associations with product-level evidence showing that these subgroups also differ in nutritional composition, nutrient profiling outcomes, energy density, and hyperpalatability.

These findings support the use of nutrient profiling, energy-density assessment, and hyperpalatability criteria as complementary tools to processing-based classification. For NCD prevention and management, the results suggest that dietary guidance should consider both the degree of processing and the specific nutritional and formulation characteristics of UPF subgroups. Future research should examine how nutrient profile, food matrix, hyperpalatability, portion size, frequency of intake, and overall dietary context jointly influence the relationship between UPF consumption and NCD outcomes.

## Figures and Tables

**Table 1:** Nutrient thresholds based on FoPL-MTL/100 grams (g) of solid products.

Nutrients of Concern	Green (Low)	Amber (Medium)	Red (High)
Total fats	≤3 g	>3 g to ≤17.5 g	>17.5 g
Saturated fats	≤1.5 g	>1.5 g to ≤5 g	>5 g
Sugars	≤5 g	>5 g to ≤22.5 g	>22.5 g
Salt	≤0.3 g	>0.3 g to ≤1.5 g	>1.5 g

**Table 2:** Nutrient thresholds based on FoPL-MTL/100 mL (milliliter) of liquid products.

Nutrients of Concern	Green (Low)	Amber (Medium)	Red (High)
Total fats	≤1.5 g	>1.5 g to ≤8.75 g	>8.75 g
Saturated fats	≤0.75 g	>0.75 g to ≤2.5 g	>2.5 g
Sugars	≤2.5 g	>2.5 g to ≤11.25 g	>11.25 g
Salt	≤0.3 g	>0.3 g to ≤0.75 g	>0.75 g

**Table 3:** Main UPF groups and subgroups according to their reported associations with NCD risk.

Group	Main Food Group	Subgroups
*Group 1:* UPFs Reportedly Associated with Increased Health Risks	Beverages	*Artificially sweetened beverages, Sweetened beverages*
	Composite Meals	*One-dish Meal ^1^,*
	Fats and Sauces	*Dressings and sauces, Vegetable margarines, Spread*
	Meat	*Processed Meat*
	Snacks	*Salty and Fatty snacks, Pastries, Sweets ^2^*
	Dairy Products:	*Dairy Dessert, Ice cream*
*Group 2:* UPFs Reportedly Associated with Lower Health Risks	Beverages	*Plant-based milk substitute*
	Plant-based alternatives	*Plant-based alternatives products ^3^*
	Cereal Products:	*Bread, Breakfast Cereals, Other Cereal Products ^4^*
	Snacks	*Biscuits and Cakes, Chocolate snacks*
*Group 3:* UPFs with No Reported Associations with Health Risks	Beverages:	*Coffees, Unsweetened Beverages*
	Cereal Products:	*Potato/potato-based products*
	Composite Meals:	*Pizza and quiches, Sandwich*
	Fruits and Vegetables:	*Dried Fruit, Vegetable based soup, Processed fruits and vegetables*
	Meat	*Fish and Seafood, Beef, Poultry*
	Dairy Products	*Cheese, Yogurt*
	Snacks	*Nuts, Appetizers ^5^*

^1^ Complete meals consisting of multiple ingredients combined into a single dish and intended to be consumed as a main meal; ^2^ generally refers to sugar-based confectionery products that are distinct from chocolates, pastries, biscuits, and cakes; ^3^ generally refers to products designed to replace animal-based foods using plant-derived ingredients except plant-based milk substitute; ^4^ broad category that includes cereal-based foods that do not fit into more specific cereal; ^5^ savory ready-to-eat snack products, including crackers, pretzels, popcorn, chips, breadsticks, and similar snack items not classified under nuts, sweets, chocolates, pastries, or biscuits and cakes.

**Table 4:** Distribution of nutritional factors (energy, carbohydrates and protein content) per risk group with Q1/median/Q3.

Group	Main Food Groups and Food Subgroup	Energy (kcal)	Protein (g)	Carbohydrates (g)
**Group** **1**	**Beverages** (artificially sweetened and sweetened)	16.00/38.00/63.00	0.00/0.10/2.80	3.40/7.90/11.00
	**Composite Meals** (one-dish meal)	134.00/204.00/272.00	4.90/8.10/11.00	10.00/22.00/37.00
	**Dairy** (dairy dessert and ice cream)	122.50/200.00/275.00	2.60/3.50/4.60	19.20/27.00/31.00
	**Fats and Sauces** (dressing sauces and vegetable margarines)	114.00/256.00/482.00	1.20/2.15/5.00	4.30/7.00/12.00
	**Meat and Fish** (processed meat)	139.75/235.00/316.25	15.50/19.70/25.00	0.50/0.80/1.50
	**Snacks** (salty and fatty snacks, pastries, and sweets)	256.95/398.00/495.00	1.00/5.90/7.50	45.00/53.00/61.00
**Group** **2**	**Beverages** (plant-based milk substitute)	31.00/45.00/63.00	0.50/0.80/3.00	2.50/3.20/9.00
	**Cereals** (bread, breakfast cereals)	268.00/309.00/396.00	7.60/9.40/11.40	45.00/50.60/68.00
	**Plant-based Alternatives**	185.00/214.00/239.00	8.47/13.00/16.30	7.88/13.00/17.00
	**Snacks** (biscuits and cakes, and chocolate snacks)	436.00/474.00/520.00	5.70/6.95/8.20	51.88/60.85/67.00
**Group** **3**	**Beverages** (coffees, unsweetened)	29.38/102.00/382.00	1.12/1.50/6.05	4.30/24.00/64.30
	**Cereals** (potato/potato-based)	268.75/374.00/429.00	6.50/8.40/11.00	35.00/59.50/71.00
	**Composite Meals** (pizza and quiches, and sandwich)	225.00/251.00/315.25	6.96/9.10/11.00	23.00/28.00/35.00
	**Dairy** (cheese and yogurt)	88.00/103.00/162.00	3.40/4.00/8.40	4.00/12.00/14.00
	**Fruits and Vegetables** (dried fruit, vegetable-based soup, and processed fruits and vegetables)	64.00/107.50/283.00	0.90/1.95/3.27	3.82/10.20/39.75
	**Meat and Fish** (seafood, fish, poultry)	134.00/174.00/225.00	12.00/17.00/22.00	0.50/1.70/9.45
	**Snacks** (nuts)	513.00/559.00/604.50	14.00/17.00/22.10	12.50/29.00/42.00

**Table 5:** Distribution of nutritional factors (nutrients of concern) per risk group with Q1/median/Q3.

Group	Main Food Groups and Food Subgroup	Total Fat (g)	Saturated Fat (g)	Salt (g)	Sugars (g)
**Group** **1**	**Beverages** (Artificially sweetened and sweetened)	0.00/0.00/1.00	0.00/0.00/0.50	0.00/0.01/0.10	2.60/6.70/11.00
	**Composite Meals** (one-dish meal)	3.70/6.60/11.0	0.80/1.80/3.10	0.85/1.20/1.73	0.90/1.90/3.40
	**Dairy** (dairy dessert and ice cream)	3.20/8.60/13.00	1.90/6.40/9.00	0.10/0.15/0.24	15.55/23.00/26.00
	**Fats and Sauces** (dressing sauces and vegetable margarines)	3.80/21.10/47.00	0.60/3.10/6.75	1.10/1.60/2.50	1.80/4.30/7.50
	**Meat and Fish** (processed meat)	6.00/16.00/25.00	2.20/6.00/9.70	1.90/2.20/3.90	0.00/0.50/0.80
	**Snacks** (salty and fatty snacks, pastries, and sweets	0.50/15.53/26.07	0.10/4.00/10.00	0.07/0.27/0.69	10.00/29.50/49.80
**Group** **2**	**Beverages** (plant-based milk substitute)	1.52/1.90/2.20	0.20/0.30/0.60	0.09/0.10/0.11	1.90/2.50/3.80
	**Cereals** (bread, breakfast cereals)	4.60/6.70/9.70	0.60/1.00/1.60	1.15/1.50/2.10	1.90/3.40/5.20
	**Plant-based Alternatives**	7.97/11.00/14.00	0.90/1.30/2.45	1.30/1.70/146.75	0.60/1.25/2.50
	**Snacks** (biscuits and cakes, and chocolate snacks)	16.00/21.00/29.00	3.38/8.00/16.00	0.20/0.40/0.63	21.90/27.80/37.55
**Group** **3**	**Beverages** (coffees, unsweetened)	0.25/0.80/6.25	0.00/0.30/4.95	0.02/0.11/0.42	3.42/16.00/51.50
	**Cereals** (potato/potato-based)	2.40/6.73/14.00	0.60/1.80/4.00	0.40/0.88/1.50	1.50/5.40/19.00
	**Composite Meals** (pizza and quiches, and sandwich)	7.70/10.50/15.14	2.30/3.40/4.90	0.90/1.20/1.40	1.90/2.70/4.50
	**Dairy** (cheese and yogurt)	2.15/3.70/12.20	1.40/2.50/8.30	0.10/0.13/0.77	3.50/11.00/13.00
	**Fruits and Vegetables** (dried fruit, vegetable-based soup, and processed fruits and vegetables)	0.40/1.90/5.68	0.00/0.30/0.90	0.05/0.80/2.00	0.93/4.40/35.00
	**Meat and Fish** (seafood, fish, poultry)	3.00/8.15/14.00	1.00/1.62/4.03	1.30/1.80/3.73	0.00/0.50/0.90
	**Snacks** (nuts)	30.50/40.00/48.70	3.80/5.60/7.00	1.00/1.45/1.95	5.15/5.80/13.70

**Table 6:** Distribution of nutritional factors (energy, carbohydrates and protein content) per UPF main food group with Q1/median/Q3. Kruskal-Wallis testing was performed for *p* values with all ranging from <0.001.

Main Food Groups	Energy (kcal)	Carbohydrates (g)	Protein (g)
Beverages	20.08/40.50/63.00	2.80/7.30/11.00	0.00/0.40/2.90
Snacks	383.00/453.00/514.00	48.00/57.00/65.95	4.70/6.50/8.00
Cereals	268.00/356.00/408.00	40.70/55.00/69.00	7.00/8.70/11.00
Composite Meals	162.50/234.00/285.50	14.00/25.00/36.00	5.80/8.55/11.00
Fruits and Vegetables	64.00/107.50/283.00	3.82/10.20/39.75	0.90/1.95/3.27
Dairy	93.00/120.00/224.00	7.45/13.30/21.42	3.20/3.80/7.80
Meat and Fish	137.00/197.00/278.00	0.50/1.00/2.70	14.00/18.00/24.00
Fats and Sauces	114.00/256.00/482.00	4.30/7.00/12.00	1.20/2.15/5.00

**Table 7:** Distribution of nutritional factors (nutrients of concern) per UPF main food group with Q1/median/Q3. Kruskal–Wallis testing was performed for *p* values with all ranging from <0.001.

Main Food Groups	Total Fat (g)	Saturated Fat (g)	Salt (g)	Sugars (g)
Beverages	0.00/0.10/1.50	0.00/0.00/0.60	0.00/0.03/0.10	1.90/5.90/10.70
Snacks	12.00/19.00/28.00	2.00/6.20/13.00	0.10/0.36/0.65	19.30/28.00/42.04
Cereals	3.40/6.70/12.00	0.60/1.20/3.00	0.67/1.20/1.80	1.70/4.00/12.00
Composite Meals	5.12/8.25/12.00	1.10/2.50/3.80	0.85/1.20/1.60	1.10/2.20/3.77
Fruits and Vegetables	0.40/1.90/5.68	0.00/0.30/0.90	0.05/0.80/2.00	0.93/4.40/35.00
Dairy	2.70/4.50/13.00	1.70/2.75/8.70	0.10/0.14/0.50	5.10/12.00/18.00
Meat and Fish	4.00/11.00/21.00	1.20/3.10/7.90	1.70/2.10/3.80	0.00/0.50/0.80
Fats and Sauces	3.80/21.10/47.00	0.60/3.10/6.75	1.10/1.60/2.50	1.80/4.30/7.50

**Table 8:** Frequency and percentage of Nutri-Score classifications among UPF subgroups categorized according to their reported associations with health risks.

Group	Main Food and Subgroups	Nutri-Score A	Nutri-Score B	Nutri-Score C	Nutri-Score D	Nutri-Score E
**Group 1**	**Beverages** (Artificially sweetened and sweetened)	67 (6.7%)	103 (10.4%)	247 (24.8%)	176 (17.7%)	401 (40.3%)
	**Composite Meals** (one-dish meal)	48 (9.3%)	64 (12.4%)	244 (47.3%)	139 (26.9%)	21 (4.1%)
	**Dairy** (dairy dessert and ice cream)	66 (11.3%)	21 (3.6%)	134 (22.9%)	227 (38.9%)	136 (23.3%)
	**Fats and Sauces** (dressing sauces and vegetable margarines)	27 (4.1%)	32 (4.8%)	140 (21.2%)	198 (30.0%)	263 (39.8%)
	**Meat and Fish** (processed meat)	7 (1.2%)	16 (2.7%)	73 (12.2%)	193 (32.3%)	308 (51.6%)
	**Snacks** (salty and fatty snacks, pastries, and sweets	67 (2.7%)	96 (3.9%)	270 (10.9%)	734 (29.5%)	1319 (53.1%)
**Group 2**	**Beverages** (plant-based milk substitute)	3 (1.4%)	82 (38.3%)	61 (28.5%)	36 (16.8%)	32 (15.0%)
	**Cereals** (bread, breakfast cereals)	77 (10.9%)	64 (9.1%)	293 (41.6%)	219 (31.1%)	51 (7.2%)
	**Plant-based Alternatives**	69 (29.0%)	52 (21.8%)	49 (20.6%)	44 (18.5%)	24 (10.1%)
	**Snacks** (biscuits and cakes, and chocolate snacks)	22 (0.8%)	20 (0.7%)	231 (8.0%)	944 (32.7%)	1670 (57.8%)
**Group 3**	**Beverages** (coffees, unsweetened)	0 (0.0%)	2 (7.7%)	13 (50.0%)	1 (3.8%)	10 (38.5%)
	**Cereals** (potato/potato-based)	142 (13.2%)	115 (10.7%)	335 (31.2%)	395 (36.8%)	85 (7.9%)
	**Composite Meals** (pizza and quiches, and sandwich)	10 (3.5%)	5 (1.8%)	98 (34.5%)	130 (45.8%)	41 (14.4%)
	**Dairy** (cheese and yogurt)	106 (10.2%)	163 (15.6%)	530 (50.8%)	197 (18.9%)	47 (4.5%)
	**Fruits and Vegetables** (dried fruit, vegetable-based soup, and processed fruits and vegetables)	34 (15.1%)	25 (11.1%)	86 (38.2%)	63 (28.0%)	17 (7.6%)
	**Meat and Fish** (seafood, fish, poultry)	44 (9.3%)	55 (11.7%)	127 (26.9%)	122 (25.8%)	124 (26.3%)
	**Snacks** (nuts)	1 (4.3%)	1 (4.3%)	11 (47.8%)	9 (39.1%)	1 (4.3%)

## Data Availability

The original contributions presented in this study are included in the article/Supplementary Material. Further inquiries can be directed to the corresponding authors.

## Supplementary Materials

The following supporting information can be downloaded at: https://www.mdpi.com/article/10.3390/foods15152651/s1, Table S1: Food Subgroups Included in Each Main Food Group of Ultra-Processed Food (UPF) Products; Table S2: Frequency and Percentage of Ultra-Processed Food (UPF) Products by Subgroup (N = 13,025); Table S3: Frequency and Percentage Distribution of Nutri-Score Grades (A–E) by Food Category; Table S4: Frequency Distribution of MTL Nutrient Categories in Ultra-Processed Foods Belonging to Group 1; Table S5: Frequency Distribution of MTL Nutrient Categories in Ultra-Processed Foods Belonging to Group 2; Table S6: Frequency Distribution of MTL Nutrient Categories in Ultra-Processed Foods Belonging to Group 3; Table S7: Frequency and Percentage by MTL Nutrient Classification Across Main Food Groups; Table S8: The frequencies and percentages of UPF products within each subgroup according to level of energy density; Table S9: The frequencies and percentages of UPF products Energy Density Distribution of Foods Across Different Food Groups.

## Abbreviations

The following abbreviations are used in this manuscript:

CSOD	Carbohydrate + Sodium
EFSA	European Food Safety Authority
FoPL	Front-of-pack labeling
FS	Fat + Sugar
FSAm-NPS	Food Standards Agency nutrient profiling model
FSOD	Fat + Sodium
HPF	Hyperpalatable food
MASLD	Metabolic dysfunction-associated steatotic liver disease
MedDiet	Mediterranean diet
MTL	Multiple traffic light
NCDs	Non-communicable diseases
NOVA 1	Unprocessed or minimally processed foods
NOVA 2	Processed culinary ingredients
NOVA 3	Processed foods
NOVA 4	Ultra-processed foods
ODBL	Open Database License
UPFs	Ultra-processed foods
