# Supplementary material for: Are All Ultra-Processed Foods Created Equal? Descriptive Analysis of Nutritional Composition, Nutritional Quality, Energy Density and Hyperpalatability in Relation to Non-Communicable Disease Risk
Source: Foods. 2026 Jul 28;15(15):2651. doi: 10.3390/foods15152651 (PMC13465375; doi:10.3390/foods15152651)
Supplement: Supplementary file 1 [file foods-15-02651-s001.zip › foods-4436660-supplementary.pdf]

Are All Ultra-Processed Foods Created Equal? An Assessment of Nutritional Composition, Nutritional Quality, Hyperpalatability in Relation with Non-Communicable Disease Risk

Supplementary Materials

Table S1. Food Subgroups Included in Each Main Food Group of Ultra-Processed Food (UPF) Products

Main Food Group	Subgroup
Beverages	Artificially Sweetened Beverages, Coffee, Plant-based Milk Substitute, Sweetened Beverages, Unsweetened Beverages
Cereals	Bread, Breakfast Cereals, Other Cereal Products[1], Potatoes/Potato-based Products
Composite Meals	One-dish Meals[2], Pizza and Quiches, Sandwiches
Dairy	Cheese, Dairy Desserts, Ice Cream, Yogurt
Fats and Sauces	Dressings and Sauces, Spreads, Vegetable Margarines
Fruits and Vegetables	Dried Fruit, Processed Fruit and Vegetable, Vegetable Soup
Meat and Fish	Beef, Fish, Poultry, Processed Meat and Fish, Seafood
Plant-based Alternatives	Plant-based Alternatives[3]
Snacks	Appetizers[4], Biscuits and Cakes, Chocolates, Nuts, Pastries<br>Salty and Fatty Snacks, Sweets[5]

[1] broad category that includes cereal-based foods that do not fit into more specific cereal subgroups

[2] complete meals consisting of multiple ingredients combined into a single dish and intended to be consumed as a main meal

[3] generally refers to products designed to replace animal-based foods using plant-derived ingredients.

[4] savory ready-to-eat snack products, including crackers, pretzels, popcorn, chips, breadsticks, and similar snack items not classified under nuts, sweets, chocolates, pastries, or biscuits and cakes.

[5] generally refers to sugar-based confectionery products that are distinct from chocolates, pastries, biscuits, and cakes.

Table S2. Frequency and Percentage of Ultra-Processed Food (UPF) Products by Subgroup (N = 13,025)

Main Food Group	Subgroup	n	%
**Beverages**	**Artificially Sweetened Beverages**	187	15.2
	**Coffee**	26	2.1
	**Plant-based Milk Substitute**	214	17.3
	**Sweetened Beverages**	590	47.8
	**Unsweetened Beverages**	217	17.6
	**TOTAL**	1234	100
Cereals	**Bread**	704	39.6
	**Breakfast Cereals**	461	26
	**Other Cereal Products**	45	2.5
	**Potatoes/Potato-based Products**	566	31.9
	**TOTAL**	1776	100
Composite Meals	**One-dish Meals**	516	64.5
	**Pizza and Quiches**	192	24
	**Sandwiches**	92	11.5
	**TOTAL**	800	100
Dairy	Cheese	302	18.6
	**Dairy Desserts**	153	9.4
	**Ice Cream**	431	26.5
	Yogurt	741	45.5
	**TOTAL**	1627	100
Fats and Sauces	**Dressings and Sauces**	627	95
	**Spreads**	24	3.6
	**Vegetable Margarines**	9	1.4
	**TOTAL**	660	100
Fruits and Vegetables	**Dried Fruit**	20	8.9
	**Processed Fruit and Vegetables**	172	76.4

	**Vegetable Soup**	33	14.7
	**TOTAL**	225	100
Meat and Fish	**Beef**	252	23.6
	**Poultry**	93	8.7
	**Processed Meat and Fish**	597	55.8
	**Fish and Seafood**	127	11.9
	**TOTAL**	1069	100
Plant-based Alternatives	**Plant-based Alternatives**	238	100
Snacks	Appetizers	276	5.1
	**Biscuits and Cakes**	2291	42.5
	**Chocolates Snacks**	596	11
	Nuts	23	0.4
	**Pastries**	493	9.1
	**Salty and Fatty Snacks**	125	2.3
	**Sweets**	1592	29.5
	**TOTAL**	5396	100

Table S3. Frequency and Percentage Distribution of Nutri-Score Grades (A–E) by Food Category

Main Food Group	A<br>n (%)	B<br>n (%)	C<br>n (%)	D<br>n (%)	E<br>n (%)	FSAm score<br>Q1/median/Q3.
Beverages<br>(n = 1,234)	70<br>(5.7%)	187 (15.2%)	321 (26.0%)	213<br>(17.3%)	443<br>(35.9%)	4 / 7 / 11
Cereals<br>(n = 1,776)	218 (12.3%)	178 (10.1%)	629 (35.4%)	614<br>(34.6%)	137<br>(7.7%)	3 / 9 / 13
Composite Meals<br>(n = 800)	58<br>(7.2%)	69<br>(8.6%)	342 (42.8%)	269<br>(33.6%)	62<br>(7.8%)	4 / 7 / 13
Dairy<br>(n = 1,627)	172 (10.6%)	184 (11.3%)	664 (40.8%)	425<br>(26.1%)	182<br>(11.2%)	4 / 6 / 15
Fats and Sauces<br>(n = 660)	27<br>(4.1%)	32<br>(4.8%)	140 (21.2%)	198<br>(30.0%)	263<br>(39.8%)	8 / 17 / 21
Fruits and Vegetables<br>(n = 225)	34 (<br>15.1%)	25<br>(11.1%)	86<br>(38.2%)	63<br>(28.0%)	17<br>(7.6%)	2 / 6 / 12
Meat and Fish<br>(n = 1,069)	51<br>(4.8%)	71<br>(6.6%)	200 (18.7%)	315<br>(29.5%)	432<br>(40.4%)	7 / 14 / 23
Plant-based Alternatives<br>(n = 238)	69<br>(29.0%)	52<br>(21.8%)	49<br>(20.6%)	44<br>(18.5%)	24<br>(10.1%)	0 / 2 / 11.8
Snacks<br>(n = 5,396)	92<br>(1.7%)	119<br>(2.2%)	513<br>(9.5%)	1,687<br>(31.3%)	2,985<br>(55.4%)	14 / 19 / 24

Table S4. Frequency Distribution of MTL Nutrient Categories in Ultra-Processed Foods Belonging to **Group 1**

Nutrients of Concern	MTL category	Beverages	Composite Meals	Dairy	Fats and Sauces	Meat and Fish	Snacks
Total Fat	Low	257 (88.9%)	104 (21.6%)	122 (24.4%)	123 (22.2%)	102 (17.6%)	769 (31.8%)
	Medium	21 (7.3%)	351 (73.0%)	309 (61.7%)	132 (23.9%)	204 (35.1%)	582 (24.1%)
	High	11 (3.8%)	26 (5.4%)	70 (14.0%)	298 (53.9%)	275 (47.3%)	1065 (44.1%)
Saturated Fat	Low	227 (78.5%)	209 (43.6%)	102 (20.4%)	199 (36.1%)	119 (20.5%)	873 (36.2%)
	Medium	50 (17.3%)	224 (46.8%)	107 (21.4%)	143 (26.0%)	150 (25.8%)	437 (18.1%)
	High	12 (4.2%)	46 (9.6%)	290 (58.1%)	209 (37.9%)	312 (53.7%)	1100 (45.6%)
Salt	Low	272 (95.1%)	14 (2.9%)	404 (80.2%)	38 (6.9%)	2 (0.3%)	1267 (52.5%)
	Medium	5 (1.7%)	318 (66.2%)	42 (8.3%)	236 (42.6%)	31 (5.3%)	842 (34.9%)
	High	9 (3.1%)	148 (30.8%)	58 (11.5%)	280 (50.5%)	548 (94.3%)	305 (12.6%)
Sugars	Low	72 (24.9%)	426 (88.6%)	48 (9.6%)	331 (59.9%)	576 (99.3%)	474 (19.6%)
	Medium	164 (56.7%)	55 (11.4%)	187 (37.5%)	174 (31.5%)	4 (0.7%)	458 (19.0%)
	High	53 (18.3%)	0 (0.0%)	264 (52.9%)	48 (8.7%)	0 (0.0%)	1483 (61.4%)

Table S5. Frequency Distribution of MTL Nutrient Categories in Ultra-Processed Foods Belonging to **Group 2**

Nutrients of Concern	MTL category	Beverages	Cereals	Plant-based Alternatives	Snacks
Total Fat	Low	13 (26.0%)	70 (10.4%)	6 (2.7%)	26 (0.9%)
	Medium	35 (70.0%)	575 (85.4%)	193 (86.2%)	872 (31.3%)
	High	2 (4.0%)	28 (4.2%)	25 (11.2%)	1886 (67.7%)
Saturated Fat	Low	38 (77.6%)	494 (73.4%)	133 (59.4%)	211 (7.6%)
	Medium	8 (16.3%)	142 (21.1%)	57 (25.4%)	785 (28.2%)
	High	3 (6.1%)	37 (5.5%)	34 (15.2%)	1788 (64.2%)
Salt	Low	43 (97.7%)	12 (1.8%)	1 (0.4%)	1114 (40.1%)
	Medium	1 (2.3%)	358 (53.4%)	102 (45.1%)	1608 (57.9%)
	High	0 (0.0%)	301 (44.9%)	123 (54.4%)	53 (1.9%)
Sugars	Low	25 (51.0%)	495 (73.6%)	221 (98.7%)	156 (5.6%)
	Medium	24 (49.0%)	176 (26.2%)	3 (1.3%)	668 (24.0%)
	High	0 (0.0%)	2 (0.3%)	0 (0.0%)	1959 (70.4%)

Table S6. Frequency Distribution of MTL Nutrient Categories in Ultra-Processed Foods Belonging to **Group 3**

Nutrients of Concern	MTL category	Beverages	Cereals	Composite Meals	Dairy	Fruits and Vegetables	Meat and Fish	Snacks
Total Fat	Low	7 (63.6%)	310 (30.4%)	1 (0.4%)	271 (29.2%)	128 (62.1%)	131 (29.2%)	0 (0.0%)
	Medium	2 (18.2%)	550 (53.9%)	224 (82.1%)	479 (51.7%)	57 (27.7%)	253 (56.5%)	2 (8.7%)
	High	2 (18.2%)	161 (15.8%)	48 (17.6%)	177 (19.1%)	21 (10.2%)	64 (14.3%)	21 (91.3%)
Saturated Fat	Low	7 (63.6%)	470 (46.1%)	30 (11.0%)	238 (25.8%)	173 (84.0%)	212 (47.3%)	1 (4.3%)
	Medium	0 (0.0%)	379 (37.2%)	182 (66.7%)	395 (42.9%)	22 (10.7%)	144 (32.1%)	10 (43.5%)
	High	4 (36.4%)	171 (16.8%)	61 (22.3%)	288 (31.3%)	11 (5.3%)	92 (20.5%)	12 (52.2%)
Salt	Low	7 (63.6%)	226 (22.2%)	17 (6.2%)	584 (62.8%)	74 (35.9%)	7 (1.6%)	3 (13.0%)
	Medium	2 (18.2%)	549 (53.8%)	207 (75.8%)	175 (18.8%)	57 (27.7%)	162 (36.2%)	10 (43.5%)
	High	2 (18.2%)	245 (24.0%)	49 (17.9%)	171 (18.4%)	75 (36.4%)	279 (62.3%)	10 (43.5%)
Sugars	Low	3 (27.3%)	499 (48.9%)	215 (78.8%)	305 (32.9%)	109 (52.9%)	441 (98.9%)	6 (26.1%)
	Medium	2 (18.2%)	345 (33.8%)	17 (6.2%)	618 (66.7%)	31 (15.0%)	4 (0.9%)	13 (56.5%)
	High	6 (54.5%)	177 (17.3%)	41 (15.0%)	3 (0.3%)	66 (32.0%)	1 (0.2%)	4 (17.4%)

Table S7. Frequency and Percentage by MTL Nutrient Classification Across Main Food Groups

Nutrient	Level	Beverages	Cereals	Composite Meals	Dairy	Fats and Sauces	Fruits and Vegetables	Meat and Fish	Plant-based Alternatives	Snacks
Fat	High	15 (4.3%)	189 (11.2%)	74 (9.8%)	247 (17.3%)	298 (53.9%)	21 (10.2%)	339 (32.9%)	25 (11.2%)	2972 (56.9%)
	Low	277 (79.1%)	380 (22.4%)	105 (13.9%)	393 (27.5%)	123 (22.2%)	128 (62.1%)	233 (22.6%)	6 (2.7%)	795 (15.2%)
	Medium	58 (16.6%)	1125 (66.4%)	575 (76.3%)	788 (55.2%)	132 (23.9%)	57 (27.7%)	457 (44.4%)	193 (86.2%)	1456 (27.9%)
Salt	High	11 (3.2%)	546 (32.3%)	197 (26.2%)	229 (16.0%)	280 (50.5%)	75 (36.4%)	827 (80.4%)	123 (54.4%)	368 (7.1%)
	Low	322 (94.4%)	238 (14.1%)	31 (4.1%)	988 (68.9%)	38 (6.9%)	74 (35.9%)	9 (0.9%)	1 (0.4%)	2384 (45.7%)
	Medium	8 (2.3%)	907 (53.6%)	525 (69.7%)	217 (15.1%)	236 (42.6%)	57 (27.7%)	193 (18.8%)	102 (45.1%)	2460 (47.2%)
Saturated Fat	High	19 (5.4%)	208 (12.3%)	107 (14.2%)	578 (40.7%)	209 (37.9%)	11 (5.3%)	404 (39.3%)	34 (15.2%)	2900 (55.6%)
	Low	272 (77.9%)	964 (56.9%)	239 (31.8%)	340 (23.9%)	199 (36.1%)	173 (84.0%)	331 (32.2%)	133 (59.4%)	1085 (20.8%)
	Medium	58 (16.6%)	521 (30.8%)	406 (54.0%)	502 (35.4%)	143 (26.0%)	22 (10.7%)	294 (28.6%)	57 (25.4%)	1232 (23.6%)
Sugars	High	59 (16.9%)	179 (10.6%)	41 (5.4%)	267 (18.7%)	48 (8.7%)	66 (32.0%)	1 (0.1%)	0 (0.0%)	3446 (66.0%)
	Low	100 (28.7%)	994 (58.7%)	641 (85.0%)	353 (24.8%)	331 (59.9%)	109 (52.9%)	1017 (99.1%)	221 (98.7%)	636 (12.2%)
	Medium	190 (54.4%)	521 (30.8%)	72 (9.5%)	805 (56.5%)	174 (31.5%)	31 (15.0%)	8 (0.8%)	3 (1.3%)	1139 (21.8%)

Table S8. The frequencies and percentages of UPF products within each subgroup according to level of energy density

Group	name	Low ED	Medium ED	High ED
Group1	Beverages	266 (92.7%)	12 (4.2%)	9 (3.1%)
	Composite Meals	155 (32.5%)	297 (62.3%)	25 (5.2%)
	Dairy	166 (33.2%)	328 (65.6%)	6 (1.2%)
	Fats and Sauces	189 (34.7%)	164 (30.1%)	192 (35.2%)
	Meat and Fish	168 (30.4%)	342 (62%)	42 (7.6%)
	Snacks	168 (7.1%)	1086 (45.7%)	1121 (47.2%)
Group2	Beverages	46 (93.9%)	3 (6.1%)	0 (0%)
	Cereals	1 (0.2%)	510 (76.6%)	155 (23.3%)
	Plant-based Alternatives	19 (8.4%)	207 (91.6%)	0 (0%)
	Snacks	9 (0.3%)	352 (12.8%)	2389 (86.9%)
Group3	Beverages	6 (60%)	2 (20%)	2 (20%)
	Cereals	83 (8.2%)	620 (61%)	313 (30.8%)
	Composite Meals	7 (2.6%)	239 (87.2%)	28 (10.2%)
	Dairy	674 (72.9%)	240 (25.9%)	11 (1.2%)
	Fruits and Vegetables	117 (57.4%)	71 (34.8%)	16 (7.8%)
	Meat and Fish	137 (30.8%)	289 (64.9%)	19 (4.3%)
	Snacks	0 (0%)	0 (0%)	23 (100%)

Table S9. The frequencies and percentages of UPF products Energy Density Distribution of Foods Across Different Food Groups

Food Group	Low ED	Medium ED	High ED
Beverages	318 (91.9%)	17 (4.9%)	11 (3.2%)
Cereals	84 (5%)	1130 (67.2%)	468 (27.8%)
Composite Meals	162 (21.6%)	536 (71.4%)	53 (7.1%)
Dairy	840 (58.9%)	568 (39.9%)	17 (1.2%)
Fats and Sauces	189 (34.7%)	164 (30.1%)	192 (35.2%)
Fruits and Vegetables	117 (57.4%)	71 (34.8%)	16 (7.8%)
Meat and Fish	305 (30.6%)	631 (63.3%)	61 (6.1%)
Plant-based Alternatives	19 (8.4%)	207 (91.6%)	0 (0%)
Snacks	177 (3.4%)	1438 (27.9%)	3533 (68.6%)
